# Brain-inspired spiking neural networks for decoding and understanding muscle activity and kinematics from electroencephalography signals during hand movements

**DOI:** 10.1038/s41598-021-81805-4

**Published:** 2021-01-28

**Authors:** Kaushalya Kumarasinghe, Nikola Kasabov, Denise Taylor

**Affiliations:** 1 Knowledge Engineering and Discovery Research Institute, Auckland University of Technology, AUT Tower, Level 7, cnr Rutland and Wakefield Street, Auckland, 1010 New Zealand; 2 Department of Information Technology, Faculty of Information Technology, University of Moratuwa, Katubedda, Sri Lanka; 3 School of Engineering, Computer and Mathematical Sciences, Auckland University of Technology, Auckland, 1010 New Zealand; 4 George Moore Chair in Cognitive Data Analytics, Ulster University, Derry, UK; 5 Rehabilitation Innovation Centre, Health & Rehabilitation Research Institute, Auckland University of Technology, Auckland, New Zealand

**Keywords:** Learning algorithms, Network models, Neural decoding, Brain-machine interface, Computer science, Sensorimotor processing, Electroencephalography - EEG

## Abstract

Compared to the abilities of the animal brain, many Artificial Intelligence systems have limitations which emphasise the need for a Brain-Inspired Artificial Intelligence paradigm. This paper proposes a novel Brain-Inspired Spiking Neural Network (BI-SNN) model for incremental learning of spike sequences. BI-SNN maps spiking activity from input channels into a high dimensional source-space which enhances the evolution of polychronising spiking neural populations. We applied the BI-SNN to predict muscle activity and kinematics from electroencephalography signals during upper limb functional movements. The BI-SNN extends our previously proposed eSPANNet computational model by integrating it with the ‘NeuCube’ brain-inspired SNN architecture. We show that BI-SNN can successfully predict continuous muscle activity and kinematics of upper-limb. The experimental results confirmed that the BI-SNN resulted in strongly correlated population activity and demonstrated the feasibility for real-time prediction. In contrast to the majority of Brain–Computer Interfaces (BCIs) that constitute a ‘black box’, BI-SNN provide quantitative and visual feedback about the related brain activity. This study is one of the first attempts to examine the feasibility of finding neural correlates of muscle activity and kinematics from electroencephalography using a brain-inspired computational paradigm. The findings suggest that BI-SNN is a better neural decoder for non-invasive BCI.

## Introduction

An integrated involvement of the mechanical elements of the limb and the associated neural circuitry, contribute to the execution of movements in animals. Conventional neural decoders that utilise the sensorimotor rhythms of electroencephalography (EEG) generate distinct neural commands through Event-related Synchronisation/Desynchronisation evoked as a result of moving different parts of the body. However, this results in un-naturalistic control when applied to neurorehabilitation due to the cognitive disconnection between the targeted and intended action. Development of computational models that can decode precise neuro-muscular relationships from EEG will enhance restorative Brain–Computer Interfaces for neurorehabilitation.

Several recent studies report the feasibility of extracting neuro-muscular interactions from EEG during functional upper limb movements such as grasp and lift. Pirondini et al.^1^ present a study on detecting EEG microstates in healthy participants during upper-limb reaching-and-grasping movements. The study reported the relationship between the dynamic transitions of the microstates and the upper-limb muscle activity. Yoshimura et al.^2^ present a cortical current source estimation-based approach to extract synchronised cortical activity of the brain from EEG for decoding finger movements. Artoni et al.^3^ report the feasibility of decoding neuro-muscular synergies from EEG during upper limb reaching tasks using unified independent component analysis. these studies provide promising empirical results on extracting neural signals from EEG useful to control and manipulate objects through BCIs.

Human-engineered Artificial Intelligence (AI) systems contradict what is already known about information processing in the animal brain. They cannot evolve, learn incrementally or adapt to changes in the environment, require large amounts of labelled data to train, yet can fail catastrophically even with small variations of the input. These limitations influence the development of a Brain-Inspired Artificial Intelligence (BI-AI) approaches to address the weaknesses in current AI systems. The lack of interpretability of computational models used for decoding neural activity is a major limitation in many BCIs that use less-interpretable machine learning approaches such as Support Vector Machine, Linear Discriminant Analysis, Generalised Linear Models, Independent Component Analysis, and deep Convolutional Neural Networks. These approaches result in BCIs that often behave as ‘black boxes’. They do not allow opportunities to extract new knowledge for a better understanding of the cognitive processes when interacting with the BCIs. This lack of interpretability limits the feasibility of using known knowledge of the cognitive processes in neuroscience for improving computational models.

Artificial Neural Networks (ANN), as a sub-set of AI, present mathematical and computational interpretations of neurons and neural network of the brain. Recent literature reveals several brain-inspired computational models that aim to model complex brain dynamics, leading to a better understanding of information representation and processing in the brain, and enable learning and adaptation in the computational model^4–9^. Deep Convolutional Neural Networks (CNN) are successfully applied for prediction in AI systems^10,11^; however, they result in static vector-based learning of input data. These vector-based models use fixed structures of neurons that require many layers of classic hierarchical abstraction to achieve a statistically significant accuracy. The lack of interpretability of knowledge gained through learning and the reduced capability for reasoning makes them vulnerable to failures^12^.

### Brain-Inspired Spiking Neural Networks

Spiking Neural Networks (SNN) as the third generation of ANN, more closely model the behaviour of a living nervous system as it considers both spatial and temporal aspects of input data for building the computational model. This paper presents a Brain-Inspired Spiking Neural Network (BI-SNN) model to address the previously stated limitations in the current BCI literature. BI-SNN enables precise spike timing in spiking neural populations using spike-time based learning rules and provide a promising direction for building a new type of BCI called Brain-Inspired Brain–Computer Interfaces (BI-BCI’s). The proposed BI-SNN is a generic SNN architecture that can be applied for the predictive modelling of spatio-temporal data streams such as to predict muscle activity and kinematics from EEG during various human activities. Here we show that the proposed BI-SNN approach enhances the decoding of forearm muscle activity and kinematics from EEG during grasp and lift movements.

We experimentally validated the BI-SNN model using the publicly available WAY-GAL-EEG (Wearable interfaces for hAnd function recovery Electroencephalography Grasp-And-Lift) dataset^13,14^. The dataset contains simultaneous EEG, Electromyography (EMG), force and kinematic signals recorded from 12 healthy participants during cued grasp and lift (GAL) movements. The participants performed a series of grasp and lift trials of a small object. During a GAL trial, the participant reached to the object, grasped it using the index finger and thumb, and lifted it a few centimetres up in the air, held it stably for a couple of seconds, and then replaced and released the object. An LED light cued the start and end of a GAL trial.

The dataset contained EMG signals from five sensors that monitored the muscle activity of the Anterior Deltoid (AD), Brachoradial (B), Flexor Digitorum (FD), Common Extensor Digitorum (CED) and First Dorsal Interosseous (FDI) muscles of the right arm. Data from kinematics sensors was gathered using 3D position sensors placed on the object, wrist, thumb and index finger. Each sensor recorded x,y,z position and azimuth, elevation and roll angles. The signals recorded from multiple devices were synchronised using a sync signal recorded by each device. More information about the data collection protocol can be found in^13^. The EEG signals were pre-processed to remove the EEG artefacts such as the eye blinks, vertical and horizontal eye movements and generic discontinuities using the ADJUST plugin^15^ of the EEGLab^16^. The artefact-free EEG signal was then filtered using a band-pass filter to extract the alpha, beta and gamma frequency bands and then rectified and down-sampled to 100Hz. The training dataset contained approximately fifteen grasp-and-lift trials per participant and corresponds to a total duration of 12 min.

**Figure 1:**
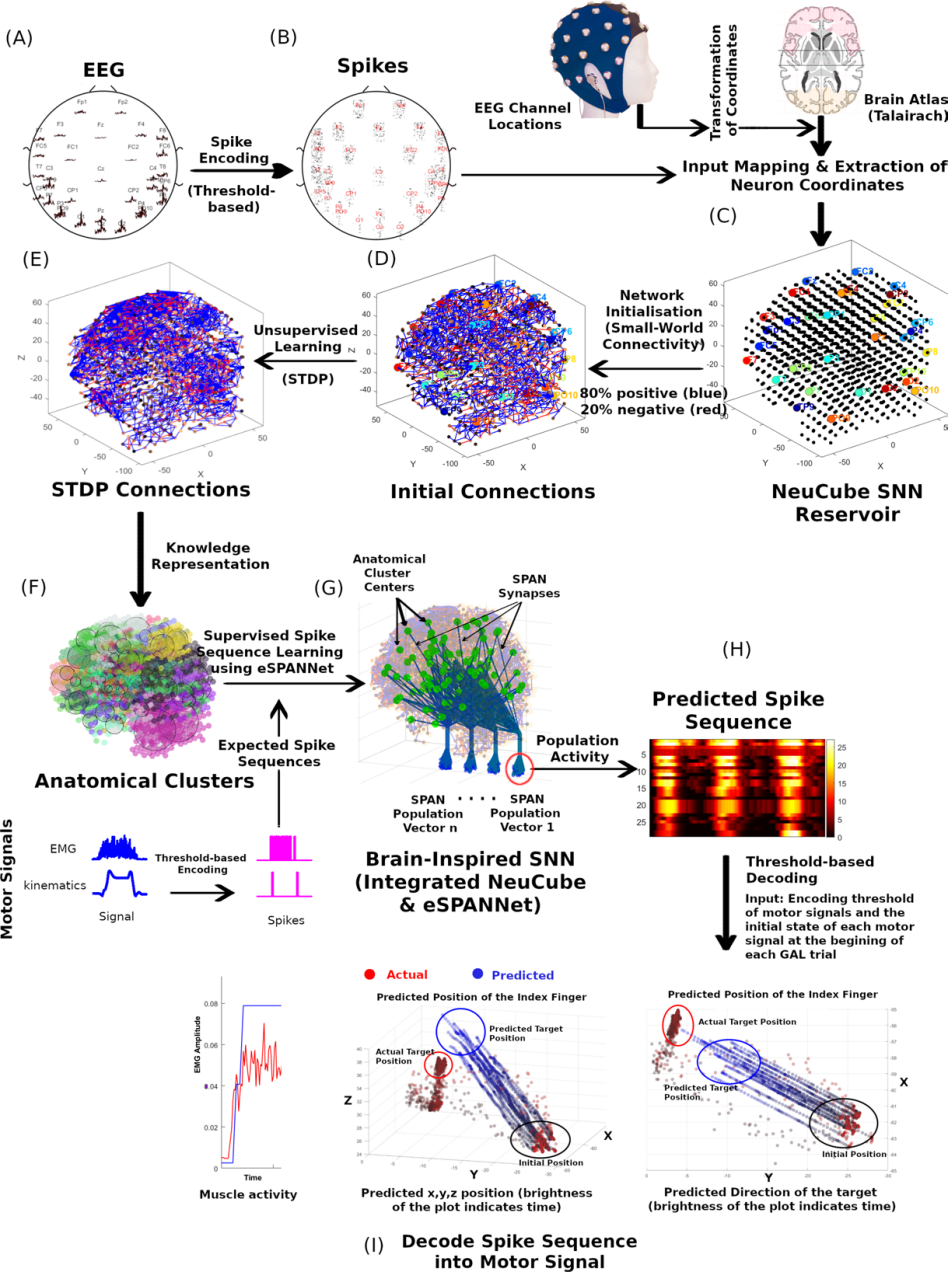
Integration of the eSPANNet with the NeuCube SNN architecture and major steps in training a BI-SNN model—(**A**) filtered EEG, (**B**) spike encoding, (**C**) extraction of brain coordinates from a brain template and mapping EEG channel locations, (**D**) initialisation of the SNN based on the small-world connectivity principle, (**E**) unsupervised spike time dependent plasticity learning, (**F**) extraction of anatomical clusters, (**G**) training population vectors using eSPANNet learning, (**H**) predicted spike sequence by the SNN, (**I**) decoding predicted spike sequences into muscle activity and kinematics using the threshold-based decoding.

We utilised the previously proposed NeuCube^4,17,18^ and the evolving Spike Pattern Association Neural Network (eSPANNet)^19^ SNN frameworks to develop the BI-SNN model. Figure 1 illustrates the architecture of BI-SNN, which integrates the NeuCube and eSPANNet SNN models and the process of training a BI-SNN model. Learning in BI-SNN includes spike encoding, input mapping, network initialisation, unsupervised learning and the extraction of anatomical clusters that represent neural activity in different regions of interest in the brain which are specific to modeling data using the NeuCube SNN framework. As shown in Fig. 1A, the BI-SNN model considers the signals from the 32 EEG channels as the input to the model, and the EMG and kinematics signals as the expected outputs from the model. In the BI-SNN, the input signals are first encoded into spike sequences (Fig. 1B) using a spike encoding algorithm, such as the threshold-based encoding^20,21^, the Bens Spikes Algorithm^22^, or the Population Rank Coding^23^. To model the motor behaviour in a spike-based interpretation, we converted both input (EEG) and expected output (EMG and kinematic) signals into spike sequences using the threshold-based encoding method.

The spiking neurons in the NeuCube reservoir are pre-structured in 3D space according to a brain atlas and initialised based on the small-world connectivity principle^24,25^. The SNN then maps the encoded spiking activity from EEG channels into this 3D space through EEG mapping as shown in Fig. 1C. The network is initialised according to the small-world connectivity principle (Fig. 1D). As the input spike trains are fed into this reservoir through input neurons, the SNN evolves based on the spike time of pre and post-synaptic neurons according to Spike-Time Dependent Plasticity (STDP) learning^26,27^ (Fig. 1E). The BI-SNN will then cluster the spiking activity based on their anatomical locations (Fig. 1F) the and apply supervised learning specific to eSPANNet, which will enable the SNN to incrementally learn the spatio-temporal association of spiking activity correspond to distinct brain regions (Fig. 1G). The eSPANNet learning model utilises the polychronisation effect of Spiking Neural Networks^28^ to decode neural activity from spatio-temporal brain data. It contains a network of Spike Pattern Association Neurons (SPAN), a spiking neuron model which can emit spikes at the desired time point^29–34^. Finally, the BI-SNN will extract the polychronising spiking neural clusters which can generate temporally associated spike sequences correlated with the predicted event as per Fig. 1H. These predicted spike sequences are decoded back to signals for predicting different motor signals such as muscle activity and kinematics by using the encoding threshold values of each motor signal and initial state of each motor signal at the beginning of the GAL trial as exemplified in Fig. 1I. A detailed description of each step related to BI-SNN is presented in the methods section.

The primary goal of this study is to evaluate the feasibility of Brain-Inspired Spiking Neural Networks to construct a novel, interpretable neural decoder which can incrementally learn to predict an upcoming movement from EEG signals. The two main objectives of the study are to predict the onset and the trajectory of a movement from EEG signals. The EMG activity of the forearm muscles are used as the expected output to train the BI-SNN for decoding movement onset from EEG signals. The kinematic signals are used as the expected output from the BI-SNN for decoding the trajectory of a movement. We will evaluate four key aspects: how accurate the prediction is, the latency of the prediction, the training speed to reach an acceptable predication, and the interpretability of the model (the degree to which a human can understand the cause of a decision made by the model). The correlation^1,35–37^ between the actual and predicted motor signal is used as a measure of prediction accuracy. The average prediction latency per single time interval in a pseudo-online experimental setup is used to evaluate the prediction latency of the model. The machine learning models were trained by gradually increasing the training dataset size to evaluate the training speed. The performance of each model at a specific training dataset size was evaluated using the same test dataset and the model performance corresponds to a particular training dataset size is used to compare the training speed. The interpretability of the BI-SNN model is evaluated using the connectivity patterns of each subject-specific BI-SNN model^38^.

The novelty of the BI-SNN approach is its ability to learn which area of the brain carry useful information for decoding a certain motor behaviour in an incremental and online manner. The brain-inspired architecture enhanced the ability to explain the reasons behind the model predictions in a form which is comprehensible to human understanding.

This study evaluated the performance of predicting the activity of the five muscle groups and 24 object and hand kinematic sensor recordings from EEG using the BI-SNN model and compared with the Generalised Linear Model (GLM) approach. GLM is used in the analysis as the main baseline method for comparison as it has been the most popular method used in the literature so far for the task under consideration. While Recurrent Neural Networks is a popular method for solving other tasks, they lack interpretability and knowledge representation of the model which is one of the main advantages of proposed BI-SNN, along with achieving a higher accuracy. GLM still offer some limited interpretability, and this is another reason to use it as a benchmark method.

## Results

The performance of reconstructing 29 motor signals was evaluated using the cross-correlation between the actual and predicted signals. The cross-correlation measured the similarity between the predicted and actual signals as a function of a short displacement of one signal relative to the other. Here we used a time lag of 100 ms for calculating the cross-correlation. For each motor signal, the cross-correlation coefficients between the actual and the predicted activity using the BI-SNN, eSPANNet and GLM approaches were obtained. Each model was trained separately using alpha, beta and gamma frequency bands of the EEG signal. The maximum coefficient of the cross-correlation sequence returned from all three frequency bands by each approach was recorded. This maximum coefficient indicates the best fit between the actual and predicted signals within a short displacement permitted by the 100 ms lag. The comparative analysis was performed using the maximum cross-correlation coefficient of each method. To interpret the results, the coefficients (0 ≤ r ≤ 1) were divided into four ranges; ‘high’, ‘moderate’, ‘weak’ and ‘very weak or no correlation’ (‘high’: r ≥ 0.7, ‘moderate’: 0.7 > r ≥ 0.5, ‘weak’: 0.5 > r ≥ 0.3, and ‘very weak’: r < 0.3).

### Deep learning in BI-SNN enhances polychronisation of SNN

The integration of eSPANNet with the NeuCube which forms the BI-SNN model, resulted in more strongly correlated population activity in comparison with the standalone eSPANNet model along with a significant biofeedback generated by the trained 3D NeuCube SNN reservior. BI-SNN demonstrated the feasibility of finding polychronising spiking neuron populations from different brain areas associated with the grasp and lift movement. The readout population activity in the BI-SNN was more temporally associated with muscle activity and hand kinematics than the pure eSPANNet. For the comparison, the normalised cross-correlation between each actual and predicted motor signal by all participants was obtained using BI-SNN, eSPANNet and GLM approaches. The average cross-correlation of each motor signal was computed using the maximum coefficient of the cross-correlation sequence correspond to each participant on that particular motor signal. Table 1 indicates the results of cross-correlation analysis using the BI-SNN (${\bar{r}}_{\text{BI-SNN}}$), eSPANNet (${\bar{r}}_{\text{eSPANNet}}$) and GLM (${\bar{r}}_{\text{GLM}}$) approaches. The highest average correlation coefficient corresponds to each motor signal is highlighted in bold text in Table 1. The comparative analysis of BI-SNN, eSPANNet and GLM showed that BI-SNN resulted in the highest average correlation between the actual and predicted motor signals in twenty-three out of the 29 motor signals. In contrast, eSPANNet corresponded to the highest average correlation in 8 out of 29 motor signals. BI-SNN resulted in the highest average correlation in majority of the motor signals that correspond to executing a grasp and lift movement. The results suggest that the BI-SNN is able to predict each individual motor signal (muscle activity and joint kinematics) more accurately than the pure eSPANNet or GLM models.

Depending on when the spike sequence learning is applied on the spike trains, the SNN models discussed in this paper can be divided into two main categories as the ‘sensor-space’ spike sequence learning models and the ‘source-space’ spike sequence learning models. A sensor-space model directly uses the spike trains extracted from sensors, such as the data obtained from EEG channels, for learning the expected spike sequences. On the other hand, the source-space spike sequence learning model first maps the spiking activity extracted from sensor data into a 3D space that represents different regions of interest in the brain. The source-space model then applies the spike sequence learning algorithm on this approximated source data to learn the expected spike sequences. The eSPANNet learning model represents a sensor-space spike model as it directly uses the spike sequences extracted from EEG sensor data for spike sequence learning. In contrast, the BI-SNN represents a source-space model as it performs the spike sequence learning on the approximated source data using the method presented in^38^ for learning the expected spike sequence. Our results show that BI-SNN, as a source-space SNN model, has the feasibility to find spiking neuron clusters that are more temporally associated with EMG activity and hand kinematics compared to the sensor-space eSPANNet model.

**Table 1:** Comparison of the average correlation coefficients between the actual and predicted motor signals by BI-SNN, eSPANNet and GLM.

Motor signal	${\bar{r}}_{\text{BI-SNN}}$	${\bar{r}}_{\text{eSPANNet}}$	${\bar{r}}_{\text{GLM}}$
Elevation of object	**0.55**	0.54	0.25
Elevation of index finger	**0.56**	0.53	0.53
Elevation of thumb	**0.57**	0.49	0.47
Elevation of wrist	**0.58**	**0.58**	0.56
Roll of object	**0.58**	0.55	0.54
Roll of index finger	**0.68**	0.65	0.67
Roll of thumb	**0.6**	0.57	0.55
Roll of wrist	**0.58**	0.52	0.53
Azimuth of object	0.55	**0**.**57**	0.5
Azimuth of index finger	**0.7**	0.68	0.69
Azimuth of thumb	**0.64**	0.62	0.59
Azimuth of wrist	**0.61**	0.58	0.58
X-position of object	**63**	0.62	0.56
X-position of index finger	**63**	0.6	0.55
X-position of thumb	0.58	**0.59**	0.52
X-position of wrist	**0.59**	0.58	0.53
Y-position of object	**0.65**	0.63	0.59
Y-position of index finger	0.58	**0.59**	0.53
Y-position of thumb	**0.58**	0.55	0.53
Y-position of wrist	**0.58**	0.54	0.53
Z-position of object	**0.6**	0.56	0.57
Z-position of index finger	**0.7**	0.68	0.66
Z-position of thumb	0.67	**0.69**	0.64
Z-position of wrist	**0.67**	0.66	0.64
Muscle-activity of AD	**0.74**	**0.74**	**0.74**
Muscle-activity of B	**0.7**	0.64	0.69
Muscle-activity of FD	0.69	**0.73**	0.67
Muscle-activity of CED	0.72	**0.73**	0.71
Muscle-activity of FDI	**0.67**	0.58	0.64

The highest average correlation coefficient corresponds to each motor signal is highlighted in bold text.

*AD* anterior deltoid, *B* brachoradial, *FD* flexor digitorum, *CED* common extensor digitorum, *FDI* first dorsal interosseous.

BI-SNN has the following features which contributed to the higher prediction accuracy by BI-SNN than the pure eSPANNet. BI-SNN has an additional hidden layer which is initially connected using the small-world connectivity principle and then evolved through STDP learning. This STDP layer enhances the evolution of polychronising spiking neural populations. In theory, it has been established that many of the functions will converge at a higher level of abstraction. So more layers will lead towards gaining better results. Further, EEG mapping transforms the spiking activity into a high dimensional space, and this type of source localisation is more compatible with neuromorphic architectures compared to other source localisation methods. The eSPANNet only process temporal information while BI-SNN process both spatial and temporal information in the model. Spatio-temporal analyses have additional benefits over purely spatial or time-series analyses with its better interpretability in terms of capturing and explaining spatio-temporal patterns of brain activity.

### Decoding continuous muscle activity in BI-SNN

The cross-correlation between the actual and predicted EMG activity shows that the BI-SNN approach results in a ‘high’ cross-correlation in predicting muscle activity of the anterior deltoid (AD), brachoradial (B), flexor digitorum (FD), common extensor digitorum (CED) and first dorsal interosseous (FDI) muscles. The Supplementary Table 1 shows the comparative analysis of predicting EMG activity using BI-SNN and GLM methods. The participant-wise mean cross-correlation coefficients in predicting all muscle activity indicate that in 9 out of 12 participants there was a ‘high’ mean correlation ($r\ge 0.7$) while in the remaining three participants there was a ‘moderate’ mean correlation ($0.7 >r\ge 0.5$). The BI-SNN delivered a ‘high’ mean cross-correlation in predicting all muscle activity. The AD muscle showed the highest average correlation of 0.74 of the group. The ‘high’ cross-correlation coefficients indicate a strong temporally associated relationship between the spiking activity of the corresponding spiking neuron population in the BI-SNN and the associated muscle activity. The cross-correlation measured the similarity between the actual and the predicted muscle activity as a function of 100 ms displacement of the predicted signal relative to the actual signal. The high correlation suggests the feasibility of accurately decoding the muscle activity from EEG signals using the BI-SNN model.

**Figure 2:**
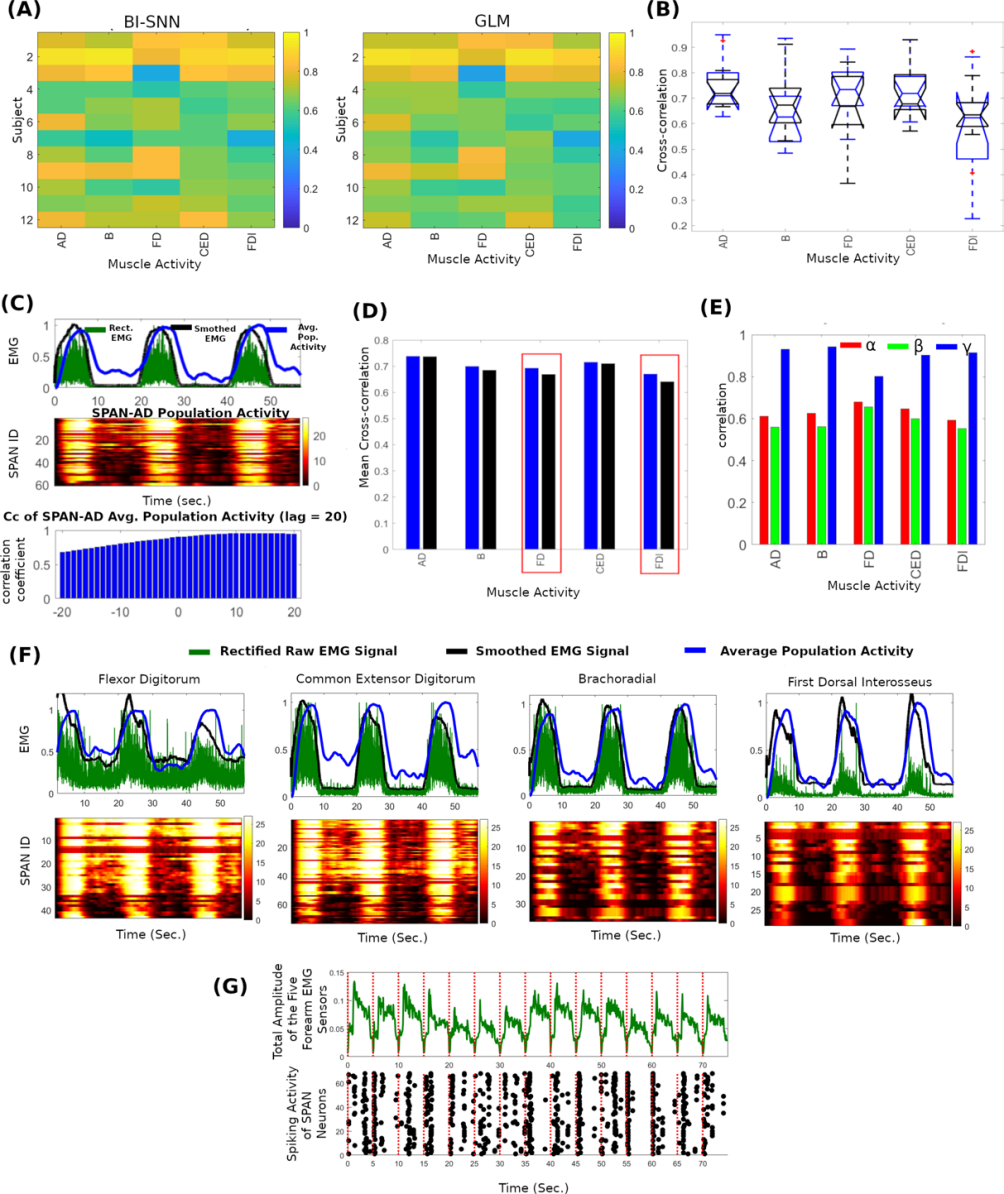
Comparative analysis of decoding muscle activity from EEG using BI-SNN and GLM approaches. (**A**) Normalised cross-correlation coefficients between the actual and predicted muscle activity from anterior deltoid (AD), brachoradial (B), flexor digitorum (FD), common extensor digitorum (CED) and first dorsal interosseous (FDI) muscles by BI-SNN and GLM methods. (**B**) Statistical distribution of the correlation coefficients by BI-SNN (blue) and GLM (black). (**C**) Calculation of the normalised cross-correlation coefficients between EMG activity of AD muscle and SPAN_AD_ population activity. Top: rectified (green) and smoothed (black) EMG signal from the AD muscle of participant 2, and the average convoluted spike sequence generated by the SPAN_AD_ (blue) population (The alpha kernel was used for spike convolution—refer Eq. (8)), middle: convoluted spike sequences emitted by spiking neurons in SPAN_AD_ population, bottom: normalised cross-correlation coefficients between the smoothed rectified EMG signal and the average SPAN_AD_ convoluted population activity using cross-correlation lag of 200 ms. (**D**) Comparison of the mean cross-correlation coefficients by the BI-SNN and GLM approaches (statistically significant differences are highlighted in red). (**E**) Band-specific cross-correlation coefficients of participant 2. (**F**) Actual muscle activity of B, FD, CED, FDI muscles and the response of corresponding SPAN population (SPAN_B_, SPAN_FD_, SPAN_CED_, SPAN_FDI_). (**G**) Population activity of SPAN_movement-onset_ and the accumulated amplitude of the five EMG sensors.

Figure 2A represents the cross-correlation coefficients between the actual and predicted muscle activity by the BI-SNN and GLM. The statistical distribution of the correlation coefficients within the participant group is presented in Fig. 2B. Figure 2C compares the mean cross-correlation of each muscle by BI-SNN and GLM. Figure 2C illustrates the effect of the change in the observed cross-correlation coefficient as a result of the permitted displacement between the two signals. A wider correlation lag of 200 ms was used to illustrate the effect. However, all results reported in this paper utilised a cross-correlation lag of 100 ms. The cross-correlation lag indicates how far the two time series are offset. The cross-correlation lag corresponds to the highest correlation coefficient represents the best fit between the two time series. At longer lags, the number of possible matches between the two signals can decrease as the longer lags increase the chance of not overlapping the two series. A short cross-correlation lag is preferred than a long lag as it indicates a shorter displacement between the signals. The shorter displacement is and indication of the probability of detecting an event within a short delay.

The comparison indicates that BI-SNN results in higher mean correlation than GLM for all muscles (Fig. 2D). Several participants such as participant 1, 2, and 3 exhibited notably higher correlation in a specific frequency band (Fig. 2E). Although such a difference was not common for all participants in the considered frequency bands, this observation suggests that muscle activity related neural information may be prominent in certain subject-specific sub-frequency bands and needs to be further investigated. Figure 2F depicts the muscle activity of the brachoradial, flexor digitorum, common extensor digitorum and first dorsal interosseous muscles of participant 2 and the population activity of the corresponding SPAN_B_, SPAN_FD_, SPAN_CED_, and SPAN_FDI_ neural populations. Figure 2G shows the total amplitude of the smoothed EMG signals from the five muscles (top) and the spike response of the SPAN_movement-onset_ population. The SPAN_movement-onset_ population was trained to produce spikes at the movement onset (bottom). In general, the SPAN_movement-onset_ population activity was synchronised with movement onset events denoted by the muscle activity, and indicate the feasibility of detecting movement onset using the proposed BI-SNN approach. However, on certain occasions, the spiking events were not closely aligned with the movement onset moment. This inconsistency can be due to multiple reasons. In addition to the movement onset, spiking events were also observed when the participant releases the object. This may be due to the activation of the same fore-arm muscle synergies that involved in both occasions. The inconsistency may also be due to the variability of the motor planning time and effort as the person becomes familiar with the task after repeating multiple trials. In addition, noise and non-stationarity of EEG have also contributed for the uncorrelated spiking events. However, the spiking behaviour of the readout population was generally correlated with the movement onset time in many grasp and lift trials.

### Accurate decoding of kinematic signals in a BI-SNN

**Figure 3:**
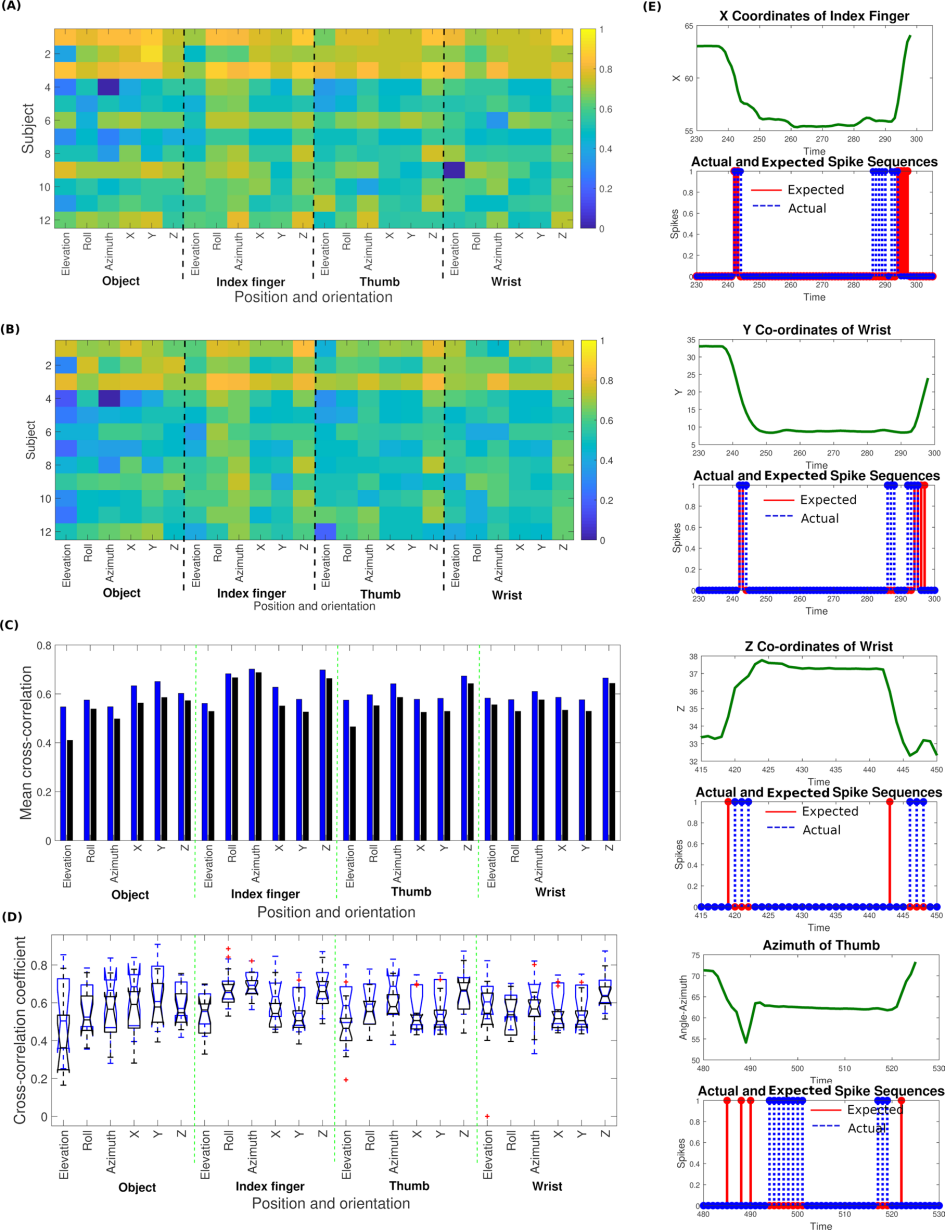
Results of predicting the object and hand kinematics using BI-SNN and GLM approaches. (**A**) The maximum coefficient of the cross-correlation sequence between the actual and predicted kinematic signal by BI-SNN. (**B**) The maximum cross-correlation coefficients between the actual and predicted kinematic signal by GLM. (**C**) Comparison between the BI-SNN and GLM methods on predicting the object and hand kinematics. (**D**) The statistical distribution of the cross-correlation coefficients within the participant group. The correlation analysis shows that the BI-SNN results in a ‘moderate’ to ‘high’ cross-correlation $(0.6 \le r \le 0.7)$ in predicting x, y, z Cartesian coordinates. (**E**) The actual kinematic signals and the spike response of a single neuron in SPAN_index-x_, SPAN_wrist-y_, SPAN_wrist-z_ and SPAN_thumb-azimuth_ populations during a Grasp-and-Lift trial.

Twenty-four kinematic sensors monitored the x, y and z position as well as the elevation, roll and azimuth angles (orientation) of the object, thumb, index finger and wrist. The BI-SNN contained separate SPAN populations trained to emit spikes according to each kinematic signal. Figure 3A,B graphically represents the cross-correlation coefficients between the actual and the predicted object and hand kinematics by the BI-SNN and GLM approaches, respectively.

BI-SNN resulted in a ‘high’ cross-correlation in predicting y coordinates of the object, z coordinates of the index finger, thumb and wrist and as well as the roll and azimuth angles of the index finger. A‘moderate’ correlation was observed in predicting x and z coordinates of the object, the x and y coordinates of the index finger, thumb and wrist, and as well as elevation, roll and azimuth of the object, thumb and wrist. Prediction of the azimuth angle and z position of the index finger showed the highest sensor-specific mean correlation (r = 0.7) within the 12 participants while participant 1 and 3 showed the highest participant-specific mean correlation of the 24 kinematics signals (r=0.8) using the BI-SNN.

Figure 3C shows a comparison of the mean cross-correlation coefficients in predicting the object and hand position by both approaches. Figure 3D shows the statistical distribution of the cross-correlation coefficients within the participant group. The comparison indicates that the BI-SNN results in a higher mean correlation compared to the GLM for all 24 kinematics signals. Figure 3E exemplifies the predicted (blue dotted line) and actual (solid red line) spike sequences during a GAL trial.

### Prediction latency of the BI-SNN

Here we report the processing time of the two SNN models, BI-SNN and eSPANNet and show their feasibility in performing real-time and online predictions. The total pseudo-online prediction latency per each participant was divided by the number of data points in the test dataset to obtain the average prediction latency per single time interval. This latency is compared with the 10 ms delay between the two consecutive input data points of the SNN model as the input signals were sampled at 100Hz sampling rate. The experiments were performed using an ordinary PC (CPU: 2.6GHz, RAM: 16GB). The source code was written in Matlab and did not utilise any parallel processing features such as multi-threading, GPU (Graphical Processing Unit) or neuromorphic computing.

**Figure 4:**
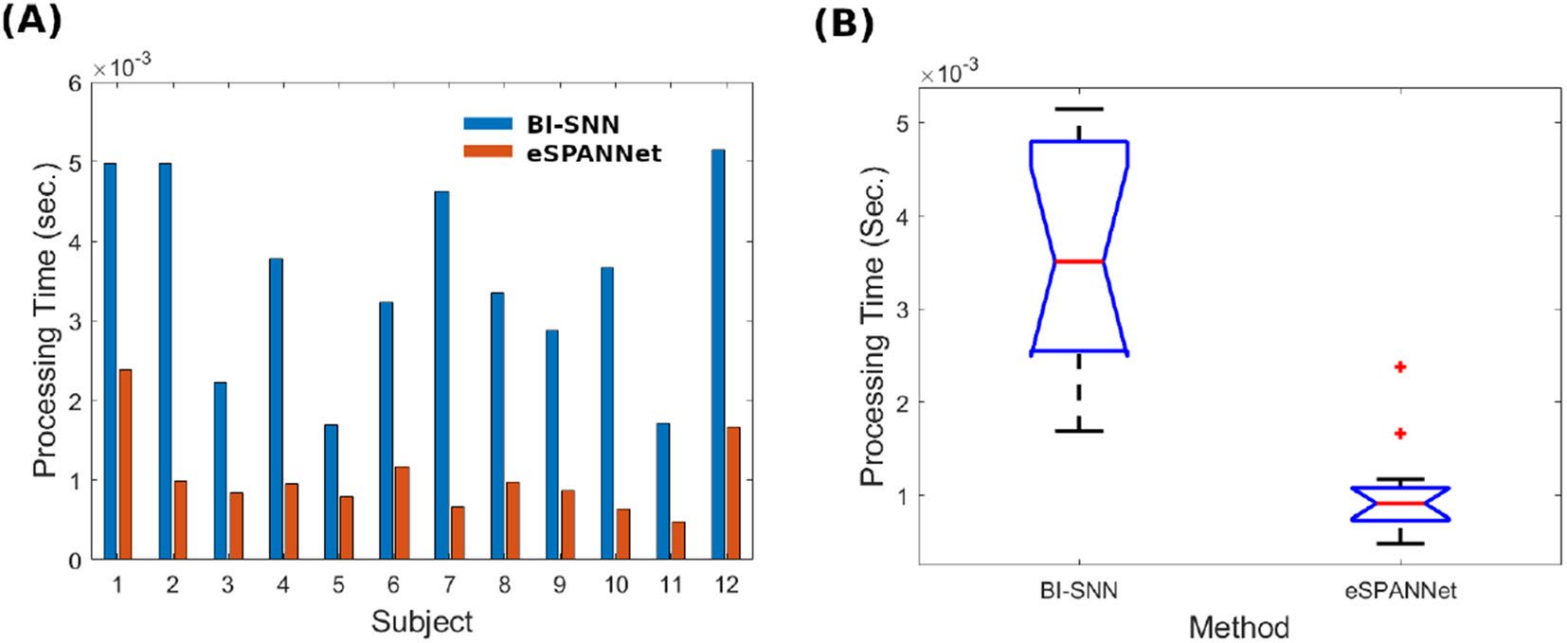
The feasibility of BI-SNN for real-time prediction. (**A**) The average prediction latency of a single time interval of the test EEG signal by each participant. (**B**) The statistical distribution of the average prediction latency within the participant group.

Figure 4A shows the average prediction time of each subject-specific SNN model to predict the spiking activity of the 29 spiking neuron populations using a single input data point of the EEG signal. Figure 4B shows the statistical distribution of the average prediction time by both methods within the group of 12 participants. The median prediction time of the 29 behavioural spike sequences by BI-SNN and eSPANNet model corresponding to a single time interval is 3.5 ms and 1 ms, respectively. The current pseudo-online system is set up to receive EEG signal at a sampling rate of 100 Hz. So, there is a 10 ms delay between two consecutive observations of the EEG in the current experimental setup. Therefore, a neural decoder which can predict the corresponding output of a single observation within this 10 ms lag will be able to perform real-time event predictions. As shown in the analysis, BI-SNN takes approximately 3.5 ms to process a single observation in a particular EEG frequency band while eSPANNet takes about 1 ms for the same task. Therefore, assuming that there are no other delays (i.e. delays in signal transmission), both spiking neuron models should be able to perform real-time predictions at a sampling rate of 100 Hz.

Although the integration of eSPANNet with NeuCube has increased the processing time, it also has improved the prediction accuracy and interpretability of the neural decoder. The trade-off between the prediction accuracy and prediction latency in BCI is a common problem and optimisation of prediction accuracy without compromising the latency of prediction and vice versa is a key challenge in BCI. The current implementations of the SNN models only utilise the sequential processing capabilities of an ordinary CPU. Such nature of processing limits the ability of real-time prediction as the spike response of each neuron is obtained in a sequential manner. The implementation of the BI-SNN and eSPANNet models in a parallel processing computational platform will enable simultaneous prediction of spiking activity of all neurons at each time-interval. Both SNN models are based on the brain-inspired computational elements and will be compatible with neuromorphic computational platforms. The induction of an appropriate parallel processing approach, such as multi-threading, multi-core processing with GPU or implementation of the SNN models in a neuromorphic chip (i.e. SpiNNaker or IBM TrueNorth) will eliminate this limitation.

### BI-SNN performance with respect to training dataset size

**Figure 5:**
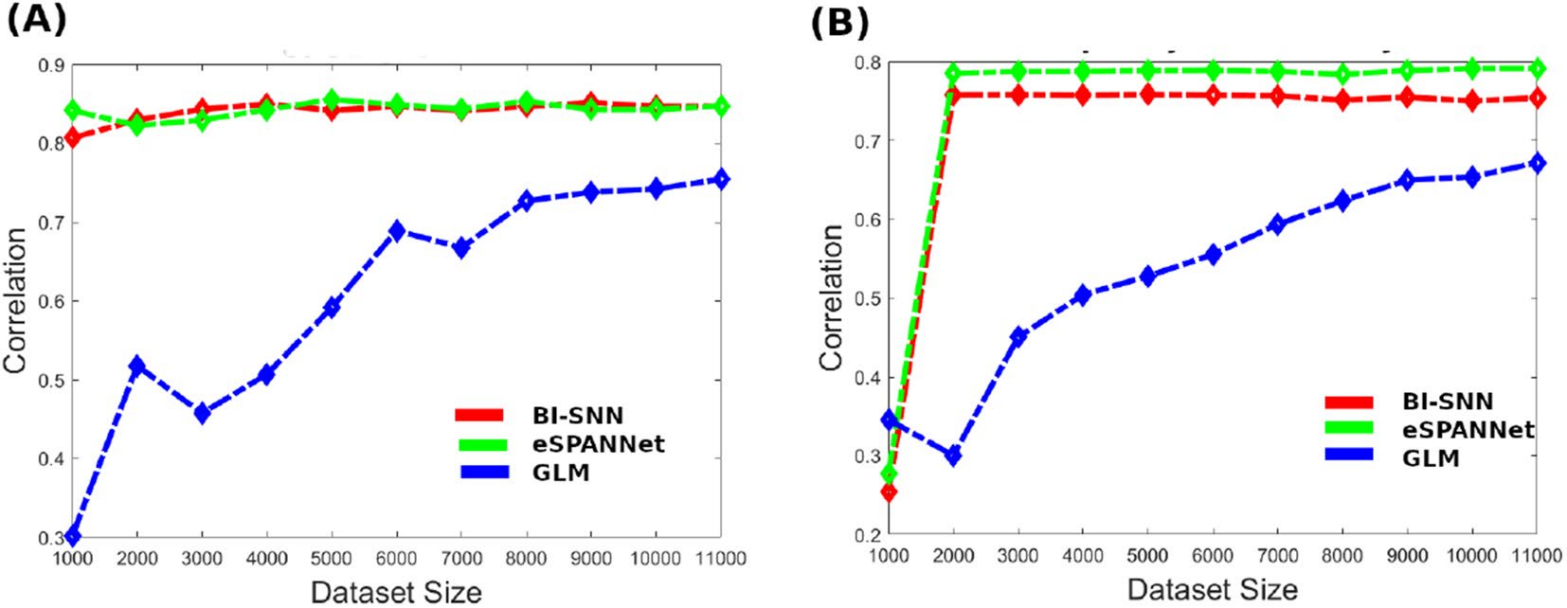
The feasibility of BI-SNN to learn from a smaller amount of training dataset. (**A**) Cross-correlation between the actual and predicted muscle activity of the flexor digitorum muscle activity with respect to the training dataset size. (**B**) Cross-correlation between the actual and predicted orientation of the index finger with respect to the training dataset size.

The analysis of model performance with respect to the dataset size suggests that the two SNN models can learn using a lesser amount of training data than GLM. Figure 5A,B exemplify the performance of the three machine learning models as a function of the training dataset size. In these two examples, the three models were able to achieve closely similar performance and we evaluated how quick each method could learn the temporal association from input spike sequences. The performance of predicting flexor digitorum muscle activity is shown in Fig. 5A. Figure 5B shows the performance of predicting the orientation of the index finger. We observed that the SNN models produce more strongly correlated output using a relatively smaller amount of training data than GLM. This ability of SNN may be due to the evolving connectionist nature of the SNN, which is also be seen in living nervous systems.

### Interpretability of the BI-SNN as a neural decoder for BCI

**Figure 6:**
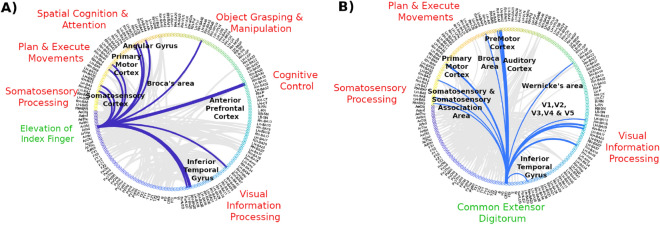
Interpretability of the subject-specific BI-SNN models. (**A**) The connectivity patterns of the BI-SNN trained using data from participant 1. The connectivity between the SPAN_index-elevation_ population vector and the spiking neurons in the 3D NeuCube SNN reservoir corresponds to different brain regions are highlighted as an exemplification. (**B**) The connectivity patterns of the BI-SNN trained using data from participant 3. The connectivity between the SPAN_CED_ population vector and the spiking neurons in the 3D SNN reservoir corresponds to different brain regions are highlighted as an exemplification.

The analysis of the connectivity in BI-SNN indicates that each SPAN population in the output layer is connected with brain areas that play a vital role in executing a grasp and lift movement. Figure 6A,B illustrate the connectivity patterns extracted from BI-SNN models of participants 1 and 3, respectively. The connectivity of a single SPAN population in each model is highlighted as an exemplification. Figure 6A shows the connectivity of the SPAN_index-elevation_ population that predict the elevation of the index finger with spiking neurons spatially located in brain regions corresponding to different Brodmann areas. Figure 6B shows the connectivity of the SPAN_CED_. The thickness of the line is proportional to the number of SPANs connected with the corresponding brain region. The connectivity pattern shows that the SPAN_index-elevation_ and SPAN_CED_ are connected with different brain areas that contribute to executing the movement such as visual information processing by the primary and secondary visual cortex, and the inferior temporal gyrus, cognitive control by the anterior prefrontal cortex, spatial cognition and attention by angular gyrus, planing and executing movement by the motor cortex and the processing of somatosensory information by the somatosensory cortex. These visualisations suggest that the BI-SNN model can contribute to a better understanding of brain activity in neurofeedback rather than a ‘black box’ or less-interpretable model.

## Discussion

This paper presents a novel brain-inspired spiking neural network model for the incremental learning of spike sequences. The proposed BI-SNN is a generic architecture that can be applied for the predictive modelling of spatio-temporal data. Here we show that BI-SNN enhances the decoding of continuous muscle activity and kinematics of upper-limb during grasp and lifting tasks. The comparative cross-correlation analysis suggests that (1) BI-SNN architecture enabled the evolution of polychronising neuron populations associated with different brain areas that contribute to the execution of the task better than the standalone sensor-space eSPANNet architecture, (2) BI-SNN reconstructed continuous muscle activity and kinematics better than the Generalised Linear Model. Further, BI-SNN demonstrated the feasibility of real-time prediction. BI-SNN achieved higher performance in reconstructing muscle activity and kinematics using a lesser amount of training data than GLM. The SNN models demonstrated the feasibility of incremental learning using the principles of evolving Connectionist Systems. BI-SNN is more interpretable for a better understanding of brain activity in neurofeedback than less-interpretable conventional machine learning models that behave as ‘black boxes’.

The scope of this study was limited to offline analysis as it is based on a publicly available dataset. Our experiments show that the proposed method allows and supports online processing as it is one of its advantages, but its application for specific tasks would require specific considerations about how this generic method can be efficiently applied. An online analysis of any time-series data generally leaves limited opportunity to understand the model behaviour or evaluate the impact of different model parameters on its performance and to optimise them accordingly. While online prediction is our final goal due to its relevance in rehabilitation interventions, to achieve that it is necessary to have a good understanding of the model behaviour. Prior knowledge of feature importance, parameters which can significantly affect the model performance, strategies for optimisation, understanding the extent to which the problem can be addressed by the proposed solution (whether it completely address the problem or if it cannot what it can and cannot achieve), the feasibility of real-time prediction, optimal sampling frequency and the interpretability of the model and its predictions are important aspects that need to be understood. This is even more challenging in single-trial event prediction from EEG signals due to the low signal-to-noise ratio and the non-stationarity of EEG. Conducting an offline analysis before the online analysis is helpful to gain a better understanding of the model parameters, their impact on model performance and the optimisation strategies. In addition, the offline analysis also lays the foundation for designing an effective data collection protocol. Therefore, the study presented in this manuscript is an offline analysis that uses a publicly available EEG dataset which is highly relevant to the specific application addressed by this research. Notwithstanding this limitation, the analysis was performed in a pseudo-online experimental setup meaning that the EEG signals were treated as they were streamed into the model for continuous and real-time prediction.

While this study did not fully confirm the possibility of real-time prediction, the average prediction time indicates that at the 100 Hz sampling rate; the SNN models can produce the corresponding output of a single input data point at a lower latency than the delay between two consecutive input data points. As we were not involved in data collection, the authors have limited knowledge about the quality of the recording.

The neural network architecture also has the following limitations. The BI-SNN utilised the Leaky Integrate and Fire (LIF) neuron model because of its computational efficiency. However, the LIF neuron model does not attempt to model the shape and the biophysical mechanisms of a spike. It considers the generation of spikes as precisely-timed events that carry the information. The LIF model only represents the timing of the spikes but not it’s shape. However, in order to accurately decode actions like reaching to grasp which occur within a very short period, the SNN should be able to represent both time and shape of a spiking event. Therefore, it would be more appropriate to use a spiking neuron model which can represent both timing and shape of a spike while keeping the computational efficiency such as the Izhikevich neuron model^39^ is more suitable than the Leaky Integrate and Fire neuron model.

The present study adds to the growing body of AI research that indicates the significance of brain-inspired models and has been one of the first attempts to examine the feasibility of finding neural correlates of muscle activity and kinematics non-invasively. The findings of the research influence the following future directions. Further studies may aim to implement the SNN models in neuromorphic processors. BI-SNN as an interpretable neural decoder, future research may investigate the feasibility of transferring the interpreted knowledge to reduce the BCI calibration time. The current study shows the importance of integrated spatial, temporal and spectral analysis of EEG to increase prediction accuracy. The proposed BI-SNN can be extended to process all three EEG bands in a single model which could be pursued in future research. The BI-SNN framework can be extended by integrating the three BI-SNN models that separately process each band-specific data in parallel where the connectivity of the SNN will guide each neuron population to receive spike sequences from a specific EEG frequency band. Future studies of the BI-SNN that utilise EEG data from motor-impaired people will support translating the technology to assistive and rehabilitation applications that improve the quality of life of motor-impaired people.

## Conclusion

This research evaluated multiple aspects of the BI-SNN including the accuracy, interpretability, prediction latency and training speed which strengthens the idea that BI-SNN is a promising approach for decoding neural activity in non-invasive brain–computer interfaces. In conclusion, the proposed BI-SNN is a potential approach to construct an interpretable neural decoder which can incrementally learn to predict complex movements in real-time from Electroencephalography signals.

## Methods

### Deep learning in brain-inspired spiking neural networks

Here we present a description of the BI-SNN model and the experimental procedure for validating and comparative analysis of the proposed BI-SNN. The ‘population vector’ model initially proposed and experimentally validated by Georgopoulos^40^ describes how the neurons in the motor cortex are trained to perform movements in different directions. Each neuron in the population demonstrated a preferred direction of movement. It was observed that the firing rate of the neurons was increased when the corresponding stimulation was presented. We utilised a similar concept in evolving spike pattern association neural network to incrementally train populations of spiking neurons to predict distinct movements by using the principles of Evolving Connectionist Systems^19^. Our previous research showed promising empirical results of the eSPANNet’s performance on predicting different upper limb movements from invasive and non-invasive brain data. So far, eSPANNet was used as a sensor-space model as it directly used the encoded spike sequence from EEG channels to evolve the population vectors of spiking neurons. Given the feasibility of the NeuCube SNN architecture to map the spiking activity into the structural and functional regions of interest in the brain^38^, we hypothesised that the integration of eSPANNet as the output layer of the NeuCube would enable better detection of polychronising spiking neural populations.

When the BI-SNN receives an input spike sequence corresponding to a particular event (i.e. flexion of the flexor digitorum muscle), the eSPANNet will first determine whether there is any SPAN in the corresponding population associated with that particular event. If there are no trained SPANs for the current event or if the trained SPANs can not produce the expected spike sequence, a new SPAN is initialised in the corresponding population vector and trained to emit the expected spike sequence at the desired time point(s). As the SNN is exposed to continuous input spike sequences associated with distinct events, BI-SNN will incrementally evolve separate SPAN populations to associate the population activity of the NeuCube reservoir with the corresponding event (see Supplementary Section 2.3 for input parameters related to eSPANNet learning). First, we briefly describe the NeuCube and eSPANNet frameworks and then present their integration for constructing the BI-SNN model.

### NeuCube brain-inspired-spiking neural network architecture

Based on the mathematical models of spiking neurons and synaptic learning, a novel evolving spatio-temporal SNN model of the brain known as NeuCube has been developed^4,17,18^. By combining anatomical and physiological information, NeuCube provides a better understanding of how activities emerge and learning happens at network level. Spike encoding, input mapping, initialisation of the SNN, unsupervised and supervised learning are the major steps in modelling and analysis of spatio-temporal data using the NeuCube SNN framework. This section briefly describes the NeuCube structure and functions (see also^18^ for further information).

#### Spike encoding

A threshold-based encoding algorithm was used to encode EEG signals in the experimental validation of the study due to its less processing time for encoding spikes. The threshold-based encoding method forms one of the simplest forms of spike encoding approaches. As a result of this simplicity, its main advantage is the ability to deliver fast encoding, which fulfils one of the requirements for the real-time information processing in SNNs. First, the temporal difference between the consecutive observations (*d*) in the input stream (*x*) is obtained to compute the encoding threshold (refer Eq. (1)).

1 $$\begin{aligned} d=\sum _{t=2}^{n}\left| x\left( t-1 \right) -x(t) \right| \end{aligned}$$

The mean value (*mean*) and the standard deviation (*std*) of the computed temporal differences are calculated to compute the encoding threshold as per Eq. (2) where *c* denotes a pre-defined variable called encoding factor.

2 $$\begin{aligned} th = mean(d)+ c\cdot std(d) \end{aligned}$$

The sign of the threshold value can be used to form both positive $(th_+)$ and negative $(th_-)$ encoding thresholds. However, since the Spike Pattern Association Neuron can not be trained with both positive and negative spikes together at the same time, here the polarity of the spike train is not considered in the current analysis. Any temporal difference that reach either the positive or negative threshold value is encoded as a positive spike event (see Supplementary Section 2.1 for spike encoding thresholds used for the experimental validation).

#### Input mapping and network initialisation

The reservoir of spiking neurons in the NeuCube is pre-structured in the 3D space according to a brain atlas. Each spiking neuron of the reservoir corresponds to a small 3D area of the brain (e.g. approximately 1 $\text{{cm}}^3$). The Talairach brain atlas annotates the 3-dimensional space of the brain in 1 $\text{{mm}}^3$ resolution including the hemisphere, lobe, tissue type (i.e. grey matter/white matter) and cell type (i.e. Brodmann area) of each brain region. By following this labelling, the 3D coordinates of spiking neurons are annotated with the corresponding anatomical labels. The initial synaptic connections in the SNN are initialised by assigning random weights using the small-world connectivity principle^24,25^.

#### Unsupervised learning using spike time dependent plasticity

Learning in a NeuCube framework is a two-phase process which includes the unsupervised learning followed by the supervised learning for classification or regression. Unsupervised learning in NeuCube applies spike-time based learning rules such as spike-time dependent plasticity on the input spike sequences received from input neurons.

The STDP learning rule quantifies the synaptic weight update of the presynaptic neuron *j*, $\Delta w_{j}$ according to the relative timing of the pre synaptic (i) spike arrival and the firing time of the post synaptic (j) spikes. The pre-synaptic spike arrival is denoted as $t_{j}^{f}$ and the firing time of the post-synaptic neuron is indicated by $t_{i}^{n}$ where $f = 1,2,3\ldots$. The total synaptic weight update $\Delta w_{j}$ is given by Eq. (3) where *W*(*x*) denotes the STDP learning window given by the Eq. (4).

3 $$\begin{aligned} \Delta w_{j}= & {} \sum _{f=1}^{N}\sum _{n=1}^{N}W\left( t_{i}^{n} - t_{j}^{f} \right) \end{aligned}$$

4 $$\begin{aligned} W\left( x \right)= & {} \left\{ \begin{matrix} A_{+} exp\left( -x/\tau _{+} \right) &{} \mathbf {for} &{} x>0\\ -A_{-} exp\left( x/\tau _{-} \right) &{} \mathbf {for} &{} x<0 \end{matrix}\right. \end{aligned}$$

The learning process results in evolving synaptic connections in the reservoir based on the relative timing of the spiking activity between pre and post-synaptic neurons. The STDP learning causes Long-Term Potentiation (LDP) when a spiking neuron receives repetitive pre-synaptic spikes arriving at a few discrete time intervals prior to the post-synaptic spikes. The repetitive spikes appear few discrete time intervals after the post-synaptic spike results in Long-Term Depression (LTD). The NeuCube framework utilises a modified version of the STDP learning rule to generate an evolving connectionist structure from input spike sequences^41^. In contrast to the conventional STDP learning, the modified STDP used in the NeuCube updates the synaptic weights between pre- and post-synaptic neurons only when a spiking neuron emits a spike. The synapses are not updated when a neuron receives a spike. Further, when a neuron fires, the modified STDP learning rule updates both pre- and post-synaptic connections. The STDP learning permits the spiking neurons in the NeuCube reservoir to associate temporally correlated input spike sequences and then transform them into a meaningful output. (see Supplementary Section 2.2 for input parameters related to network initialisation and STDP learning).

#### Supervised learning

The supervised learning obtains the spike response of the NeuCube reservoir after STDP learning for classification or regression through dynamic evolving Spiking Neural Network (deSNN) algorithm^42^. The spike response of the reservoir can be approximately map to different regions of interest in the brain through the knowledge representation framework of NeuCube^38^. The proposed BI-SNN replaces the deSNN classifier in the generic NeuCube framework by the evolving spike pattern association neural network model. the integration of eSPANNet as the output layer of NeuCube SNN architecture enables incremental learning of spike sequences to associate temporally correlated spiking activity from distinct brain areas with the output neuron layer of the BI-SNN. The next section briefly describe the eSPANNet learning algorithm.

### Evolving spike pattern association neural network

The eSPANNet^19^ is an evolving feed-forward spiking neural network model which extends the spike pattern association neuron model proposed in^29–34^. The SPAN neuron model was extended and combined with a computational interpretation of a ‘population vector model’ to derive a biologically plausible model of motor learning and adaptation in the proposed evolving Spike Pattern Association Neural Network architecture.

SPAN is a spiking neuron model which can learn to associate arbitrary spike trains allowing the processing of spatio-temporal information encoded in the precise temporal order of spikes. It is based on the spike based interpretation of the Widrow–Hoff/Delta learning rule that uses the training error as an input for its objective function for training. This training error is defined as the difference between the expected and actual output produced after each training iteration. During learning the weights are updated in such a way that it reduces the training error.

For a labeled training dataset with *n* number of class labels and, *x* number of input channels (features), a feed-forward SNN is formed to derive the synaptic weight, *w* by the proposed supervised learning model. During the time period of $\Delta t$ from $t_{1}$ to $t_{2}$, prediction of the output class label $l _{\Delta t}$ from testing spike sequences is performed using the activation time-course of $n$ readout neurons in the output layer given by $q_{n,t}$. Each readout neuron exhibits a binary state space; $Q_{readout} = \left\{ 0, 1 \right\}$, determined by the average post-synaptic spike pattern of the group of readout neurons $q_{n,t}$ during the $\Delta t$ time period.

5 $$\begin{aligned} \begin{aligned} l _{\Delta t} = f \left( q_{n,t} \right) \end{aligned} \end{aligned}$$

$q_{n,t}$ is $n$ by 1 dimension vector that represent the current spiking state (spike or no spike) of $n$th readout neurons at a given time. Using a suitable synaptic learning method, the SNN derives the synaptic weight between input and output spiking neuron layers so that for a given input spike pattern, the SNN will learn to emit a desired spike sequence by the corresponding output neuron.

The input layer contains *x* number of input neurons that feed input spike trains into the hidden layer. The hidden layer contains groups of SPAN’s arranged as *n* number of population vectors. The network architecture connects each neuron of a particular SPAN population with only one input neuron. Each SPAN is trained using a single training spike sequence received through the corresponding input neuron(s) and validated using the other training and validation spike samples during incremental learning. The output layer contains *n* number of integrate and fire neurons each associated with the corresponding population vector. Each SPAN that belong to a certain population vector is connected with only one output neuron which receive spikes through all SPANs in that particular SPAN population vector. Each neuron in the output layer acts as a readout neuron where the corresponding class label is predicted according to the behaviour of these readout neurons.

#### Incremental learning

The state of the $n$th readout neuron during $\Delta t$ time period is calculated using $\bar{I_n}$, the synaptic current from the hidden layer neurons of the $n$th population vector to $n$th readout neuron, $F_{n,t }$ postsynaptic spike pattern of the $n$th population vector during $\Delta t$ and, $th_{n}$ the firing threshold of the $n$th readout neuron.

6 $$\begin{aligned} \begin{aligned} q_{n,t} = g\left( {\bar{I}}_{n,t}, th_{n} \right) = \left\{ \begin{matrix} 1 &{} if &{} {\bar{I}}_{n,t}\ge th_{n}\\ 0 &{} otherwise &{} \end{matrix}\right. \end{aligned} \end{aligned}$$

The average synaptic current from the $n$th population vector to the $n$th readout neuron at time *t*, $\bar{I_{n}(t)}$, is given by synaptic weight $w_{m,n}$ between $m$th hidden neuron in the $n$th population vector and the convoluted spike pattern of $m$th hidden neuron in the $n$th population vector at time *t*, ${\tilde{s}}_{m,n}$,

7 $$\begin{aligned} \begin{aligned} {\bar{I}}_{n}(t)= \frac{1}{m}\sum _{i=1}^{m} I_{m,n} = \frac{1}{m}\sum _{i=1}^{m} w_{m,n} \odot {\tilde{s}}_{m,n}(t) \end{aligned} \end{aligned}$$

The convoluted spike pattern for $m$th neuron in the $n$th population vector $({\tilde{s}}_{m,n}(t))$ is obtained using a kernel function which convolute the discrete spike sequences into a continuous signal. Similar to previous studies on the spike pattern association neuron model^29^, this research applied the $\alpha$ kernel for spike convolution. If $F_{m,n}$ denotes the set of firing times of the $m$th neuron in $n$th population vector, the convoluted spike pattern $({\tilde{s}}_{m,n}(t))$ is obtained by applying the $\alpha$-kernel as per Eq. (8).

8 $$\begin{aligned} \begin{aligned} {\tilde{s}}_{m,n} (t) = \sum _{t_{m,n}^{f}\in F_{m,n}} \alpha (t-t^{f}) \end{aligned} \end{aligned}$$

The $\alpha$ kernel is defined as Eq. (9) where $\tau _{s}$ denotes the synaptic time constant that characterise the exponential decay of the convoluted spike sequence and $\Theta \left( t \right)$ represents the Heaviside step function^30^.

9 $$\begin{aligned} \alpha \left( t \right) = e\; \tau _{s}^{-1}\; t\; e^{-t/\tau _{s}}\; \Theta \left( t \right) \end{aligned}$$

$\tau _{s}$ characterises the exponential decay of a convoluted spike.

The hidden layer (*m*) contains groups of SPAN’s arranged as *n* number of population vectors. Firing times of the $m$th neuron in the $n$th population vector $(F_{m,n})$ is obtained using the post-synaptic spike pattern of the neuron $(s_{m,n})$ using the synaptic weight between input and hidden layer neurons $(w_{i,m})$, input spike train from the input neuron $(x_{i})$ and the firing threshold of the SPANs in the hidden layer $(th_{m,n})$. Therefore, the synaptic current of the neuron *m* in the SPAN population $I_{m} \left( t \right)$ is obtained using the weighted convoluted spike pattern received by the neuron *m* from the input neuron *i* as per Eq. (10).

10 $$\begin{aligned} \begin{aligned} I_{m} \left( t \right) = \sum _{i}w_{i,m}\sum _{f}\alpha \left( t - t_{i}^{f} \right) \end{aligned} \end{aligned}$$

Here, $f_{m}(t)$ denotes the firing times of the $m$th SPAN in the hidden layer marked by the time intervals its membrane potential $(I_{m}(t))$ reach the firing threshold $th_{m}$ of the neuron as per Eq. (11).

11 $$\begin{aligned} \begin{aligned} f_{m}(t) = g\left( I_{m} (t), th_{m} \right) = \left\{ \begin{matrix} 1 &{} if &{} I_{m} (t)\ge th_{m}\\ 0 &{} otherwise &{} \end{matrix}\right. \end{aligned} \end{aligned}$$

$w_{i,m}$ is calculated using the synaptic learning rule of the spike pattern association neuron^29^ as per Eq. (12).

12 $$\begin{aligned} \begin{aligned} \Delta w_{i,m} = \lambda \left( \frac{e}{\tau } ^{2} \left[ \sum _{g} \sum _{f} \left( \left| t_{i}^{f} - t_{d}^{g} \right| +\tau \right) e ^{-\frac{\left| t_{i}^{f} - t_{d}^{g} \right| }{\tau }} \right. \right. \\ \left. \left. - \sum _{h} \sum _{f} \left( \left| t_{i}^{f} - t_{out}^{h} \right| +\tau \right) e ^{-\frac{\left| t_{i}^{f} - t_{out}^{h} \right| }{\tau }} \right] \right) \end{aligned} \end{aligned}$$

where $\lambda$ is the learning rate, $\tau$ is the time constant of the kernel function, $t_{i}$, $t_{d}$ and $t_{out}$ are the times of input, desired and acutal spikes. *f*, *g* and *h* denotes the indexes of input, desired and actual spikes.

As the SPAN readout populations are evolved, each spiking neuron in the SPAN population is validated using their ability to emit spikes at desired time points for unseen input spike sequences. The BI-SNN only considers the spiking activity of the SPANs, which result in higher accuracy than a predefined threshold level. The threshold level is determined by the number of SPANs in the corresponding population and the maximum prediction accuracy of the SPANs in the population. For each unseen input spike trains, the spike response of each SPAN population will be obtained to determine the corresponding class label. Different strategies such as the average population activity or majority voting can be followed to determine the class label of a particular input spike sequence.

## Supplementary information

Supplementary Information.

## Data Availability

This research used a publicly available WAY-GAL-EEG (Wearable interfaces for hAnd function recovery Electroencephalography Grasp-And-Lift) dataset^14^ for the experimental validation of the proposed method. The dataset can be downloaded from http://dx.doi.org/10.6084/m9.figshare.988376
