# Supplementary material for: Brain-inspired spiking neural networks for decoding and understanding muscle activity and kinematics from electroencephalography signals during hand movements
Source: Sci Rep. 2021 Jan 28;11:2486. doi: 10.1038/s41598-021-81805-4 (PMC7844055; doi:10.1038/s41598-021-81805-4)
Supplement: Supplementary file 1 — Supplementary Information. [file 41598_2021_81805_MOESM1_ESM.pdf]

Brain-Inspired Spiking Neural Networks for Decoding and Understanding Muscle Activity and Kinematics from Electroencephalography Signals during Hand Movements - Supplementary Information

Kaushalya Kumarasinghe[1,*], **Nikola Kasabov**[1], **and Denise Taylor**[2]

[1]Knowledge Engineering and Discovery Research Institute, Auckland University of Technology, New Zealand
[2]Health and Rehabilitation Research Institute, Auckland University of Technology, New Zealand
[*]kaushalya.kumarasinghe@aut.ac.nz

1 Supplementary Table 1: Cross-correlation coefficients between the actual and predicted muscle activity

Table 1. Cross-correlation coefficients between the actual and predicted muscle activity (The maximum cross-correlation coefficients from the alpha, beta and gamma frequency bands are shown)

2***Subject**	**AD**		**B**		**FD**		**CED**		**FDI**	
	BI-SNN	GLM	BI-SNN	GLM	BI-SNN	GLM	BI-SNN	GLM	BI-SNN	GLM
1	0.77	0.74	0.73	0.74	0.86	0.84	0.86	0.82	0.75	0.71
2	0.93	0.93	0.94	0.91	0.8	0.77	0.9	0.93	0.92	0.88
3	0.8	0.77	0.85	0.8	0.4	0.37	0.84	0.81	0.82	0.79
4	0.62	0.69	0.62	0.7	0.54	0.55	0.62	0.67	0.65	0.66
5	0.62	0.67	0.68	0.69	0.71	0.66	0.59	0.6	0.63	0.59
6	0.81	0.78	0.66	0.65	0.71	0.67	0.61	0.57	0.68	0.64
7	0.57	0.67	0.5	0.53	0.62	0.65	0.62	0.68	0.41	0.41
8	0.72	0.68	0.62	0.6	0.84	0.81	0.66	0.66	0.58	0.56
9	0.83	0.81	0.81	0.74	0.83	0.8	0.66	0.66	0.63	0.64
10	0.72	0.7	0.6	0.59	0.56	0.57	0.67	0.66	0.66	0.63
11	0.67	0.67	0.67	0.66	0.74	0.7	0.71	0.69	0.63	0.59
12	0.8	0.74	0.72	0.61	0.72	0.63	0.84	0.77	0.68	0.61
**Mean**	0.74	0.74	0.7	0.69	0.69	0.67	0.72	0.71	0.67	0.64
**p-value**	0.957		0.322		**0.026**		0.646		**0.001**	

Key: Anterior Deltoid (AD), Brachoradial (B), Flexor Digitorum (FD), Common Extensor Digitorum (CED), First Dorsal Interosseous (FDI)

2 Input parameters used for the experimental validation

2.1 Spike Encoding Threshold

Table 2. Spike Encoding Threshold

participant id	alpha	beta	gamma
1	0.1	0.4	0.2
2	0.05	0.01	0.05
3	0.1	0.3	0.2
4	0.2	0.5	0.2
5	0.1	0.5	0.2
6	0.1	0.5	0.3
7	0.01	0.5	0.1
8	0.3	0.3	0.1
9	0.25	0.5	0.1
10	0.15	0.4	0.1
11	0.1	0.3	0.1
12	0.1	0.3	0.2

2.2 Spike Time Dependent Plasticity Learning

Small world radius = 2.5
Number of spiking neurons in NeuCube SNN = 1543
STDP learning rate = 0.1
Firing threshold on spiking neurons= 0.5
Potential leak rate = 0.002
Refactory time = 6
Probability of Long Distance Connectivity (LDC) = 0
LDC initial weight = 0.05
Number of training rounds = 1

2.3 Spike Pattern Association Neuron Learning

Sampling rate = 100
Number of time intervals per second = 10
Threshold of anatomical cluster = 0.05
Threshold of spike rate= 0.11
Time constant = 1
Number of epochs for training = 1000;
Expected spike time = 11
Learning rate = 1000000
Maximum error = 2

2.4 Leaky Integrate and Fire Neuron

Resting membrane potential = -70
Firing threshold= 100
Reset voltage after spike= -75
Membrane resistance = 0.001
Membrane time constant= 1

2.5 Evolving Spike Pattern Association Neural Network (eSPANNet) Learning

Window size = 20
Accuracy threshold for SPAN selection= 0.95

Neuron count threshold for SPAN selection = 0.9

3 List of Abbreviations

AD : Anterior Deltoid
AeInd : Elevation angle of Index finger
AeObj : Elevation angle of Object
AeThu : Elevation angle of Thumb
AeWri : Elevation angle of Wrist
AI : Artificial Intelligence
ANN : Artificial Neural Networks
ArInd : Roll angle of Index finger
ArObj : Roll angle of Object
ArThu : Roll angle of Thumb
ArWri : Roll angle of Wrist
AzInd : Azimuth angle of Index finger
AzObj : Azimuth angle of Object
AzThu : Azimuth angle of Thumb
AzWri : Azimuth angle of Wrist
B : Brachoradial
BCI : Brain-Computer Interface
BI-AI : Brain-Inspired Artificial Intelligence
BI-SNN : Brain-Inspired Spiking Neural Network
BSA : Ben's Spikes algorithm
CED : Common Extensor Digitorum
CNN : Convolutional Neual Networks
deSNN : dynamic evolving Spiking Neural Network
EEG : electroencephalography
EMG : electromyography
eSPANNet : evolving Spike Pattern Association Neural Network
FD : Flexor Digitorum
FDI : First Dorsal Interosseous
GAL : Grasp and Lift
GLM : Generalised Linear Model
ICA : Independent Component Analysis
LDP : Long-term Potentiation
LED : Light-Emitting Diode
LTD : Long-term Depression
PxInd : X position of Index finger
PxObj : X position of Object
PxThu : X position of Thumb
PxWri : X position of Wrist
PyInd : Y position of Index finger
PyObj : Y position of Object
PyThu : Y position of Thumb
PyWri : Y position of Wrist
PzInd : Z position of Index finger
PzObj : Z position of Object
PzThu : Z position of Thumb
PzWri : Z position of Wrist
SPAN : Spike Pattern Association Neuron
STDP : Spike Time Dependent Plasticity
